# Compulsive Drumming Induced by Dopamine Agonists in Parkinson’s Disease: Another Aspect of Punding

**DOI:** 10.3233/BEN-129024

**Published:** 2013

**Authors:** Carmine Vitale, Luigi Trojano, Paolo Barone, Domenico Errico, Valeria Agosti, Giuseppe Sorrentino, Dario Grossi, Gabriella Santangelo

**Affiliations:** ^1^University “Parthenope”, NaplesItaly; ^2^Istituto di Diagnosi e Cura “Hermitage Capodimonte”, NaplesItaly; ^3^Department of Psychology, Second University of Naples, CasertaItaly; ^4^Neurodegenerative Center Diseases, University of Salerno, SalernoItaly

**Keywords:** Punding, Parkinson’s disease, pramipexole

## Abstract

We report the case of a man affected by Parkinson’s disease who developed an unusual, severe, repetitive behavior characterized by an irrepressible need to drum and beat percussion instruments following to the introduction of pramipexole. This compulsive behavior was not associated to a pattern of chronic inappropriate overuse of dopaminergic medication or other psychiatric symptoms. Sharing many features with other repetitive behaviors, compulsive drumming might be considered a distinct manifestation of punding in Parkinson’s disease.

## 1. Introduction

Punding refers to a heterogeneous group of repetitive and mindless behaviors that were initially observed in psychostimulant users [1]. Punding behavior has been described as 'compulsive hobbyism', and encompasses activities such as endless computer use, cleaning and tidying, gardening, collecting, repairing and/or dismantling technical equipment and sorting common objects [2,3]. Punding often reflects premorbid affinities related to work or hobbies, but is characterized by intense, inappropriate and unproductive occupation, with potentially disruptive and severe psychosocial consequences, which patients are often unaware of [3].

Punding has been associated with dopaminergic stimulation, and a correlation to total daily dose of dopaminergic drugs in Parkinson disease (PD) has been reported [3], in analogy with other compulsive behaviors such as gambling, hypersexuality, and other repetitive, purposeless behaviors [4–8]. Here we describe a PD patient who developed disabling compulsive drumming (CD) consequent to pramipexole introduction.

## 2. Case report

A 68-year-old man was diagnosed with idiopathic PD, based on a one-year history of slowness, unilateral rest tremor and micrographia. The medical history included hypertension and diabetes; there was no family history of neurological diseases. A physical examination revealed moderate parkinsonism, with hypomimia, stooped posture, bradykinesia, right side-

ISSN 0953-4180/13/$27.50 © 2013 – IOS Press and the authors. All rights reserved dominant-rigidity and rest tremor. No additional neurological signs were found. Brain MRI showed minimal diffuse atrophy. [$^{123}$I]FP-CIT-SPECT showed bilateral reduction of tracer uptake mainly involving the putamen and more marked in the left side.

Ropinirole was started (up to 8 mg daily) but soon discontinued because of nausea and vomiting (despite motor benefits were evident). Levodopa-Carbidopa was then started and titrated to 300 mg daily, with relevant clinical improvement. Three years later, the patient presented mild fluctuations of motor symptoms and concomitant mood swings. For these reasons pramipexole was added and titrated to 1 mg t.i.d. with rapid improvement of both motor and psychiatric symptoms.

Two weeks after starting pramipexole, the patient, who has been an amateur musician and singer, developed an unhealthy obsession with playing drums and percussion instruments. He experienced an irrepressible urge to play the rhythms of popular songs by beating hands and sticks on cooking battery and tableware. In subsequent months he bought an expensive set of percussion instruments and created a devoted soundproof chamber at home, gradually increasing time spent in playing drums. The patient did not realize CD was unhealthy and time consuming, and found it extremely fascinating. Even though CD was disruptive and prevented sleep and social interactions, the patient reported an increasing need to play drums to achieve "wellbeing" and a feeling of calmness. Attempts to reduce CD produced withdrawal-like symptoms: the patient felt antsy, irritated and sometimes depressed. CD continued for 6 months. Amantadine (100 mg twice daily) did not produce beneficial effect, whereas the repetitive behavior reduced moderately after quetiapine (25 mg, twice daily). Finally, withdrawal of pramipexole resulted in dramatic reduction of CD, allowing the patient returning to his usual daily activities.

A formal neuropsychological evaluation was administered one month after onset of CD. At that time the patient was cooperative and well oriented in time and space. He achieved a normal age- and education-adjusted score on Mini Mental Examination and normal scores on tasks assessing frontal lobe functions, spatial short-term memory, visuospatial and linguistic abilities (Table 1). The patient only showed impaired performance on tests assessing verbal long-term memory and selective attention.

He was not affected by major depressive disorders according to DSM IV criteria and his total score on the Hamilton Depression Rating Scale was low [4]. He did not present visual hallucinations or other psychotic symptoms and ICDs as disclosed by both Neuropsychiatric Inventory Scale (NPI) and Minnesota Impulsive Disorders Interview (MIDI), respectively. A clinical interview showed that the patient fulfilled clinical criteria for punding, as previously reported [1–3,9].

Table 1
Formal neuropsychological testing

| Cognitive tests | Score | Equivalent score* |
|---|---|---|
| MMSE | 27/30 | |
| *Memory* | | |
| Immediate recall | 34 | 2 |
| Delayed Recall | 4 | 0 |
| Verbal span | 4 | 2 |
| Spatial span | 4 | 1 |
| ROCF-delayed recall | 18.5 | 4 |
| *Frontal functions* | | |
| ROCF-copy | 32 | 3 |
| Phonological fluency | 23 | 1 |
| Attentive matrices | 35 | 0 |
| Trail making test: part B | 272 | 1 |
| Trail making test: part B-part A | 198 | 1 |
| Stroop test: interference | 10 | 1 |
| *Visuospatial functions* | | |
| Benton Judgment Line Orientation Test | 20 | |

ROCF, Rey-Osterrieth complex figure test. *Note: where available equivalent scores refer to Italian age- and education-adjusted norms: 0 means below the normal range; 1 to 3 mean within the normal range, at about the 10th, 25th and 50$^{th}$ centile, respectively.

## 3. Discussion

Punding is a relatively uncommon but underreported behavioral feature of PD characterized by stereotyped, repetitive, purposeless, and disruptive behavior accompanied with a form of fascination related to ongoing activities.

In the absence of other features of dopamine dysregulation syndrome (DDS), our patient developed a peculiar, stereotyped and compulsive behavior characterized by an urge to repeatedly play drums and percussion instruments, in the setting of mild fluctuating PD. Sharing many features with other compulsive behaviors, CD might be proposed as a previously unreported manifestation of punding in PD. CD can be conceptualized as a multidimensional maladaptive pattern of repetitive and mindless behavior, leading to clinically significant impairment, as manifested by need for increased amounts of repetitive playing sessions, characteristic withdrawal symptoms (e.g., anxiety), need to spend increasing time in playing drums to achieve calmness and wellbeing, unsuccessful efforts to cut down, reduction in other activities, and maintenance despite persisting/recurring psychological and social limitations caused or exacerbated by the repetitive behavior.

Apart from compulsive drumming, our patient did not show other features of DDS or prior psychiatric conditions. He showed impairments on tests assessing verbal long-term memory and selective attention but was not affected by global cognitive impairment or dementia, as defined by DSM-IV-TR.

It has been proposed that basal ganglia control storage and selection of specific competing motor programs [10–12]. A dysfunction of such processes controlling habitual routine behaviors may elicit the abnormal expression of stereotyped motor behaviors, and difficulty in time estimation.

In our patient, the onset of CD appeared to be related to dopamine agonists therapy. Indeed, CD developed during pramipexole treatment, and improved when the drug was discontinued, whereas the other antiparkinsonian medications were unchanged. The basis for this drug-induced pathologic behavior might be related to alterations of brain's reward and motor systems. In PD, dopamine neurons projecting to ventral striatum are less severely affected by the disease process [13]. This raises the possibility that dopamine replacement therapy in the motor striatum leads to overdosing of the limbic striatum and the occurrence of repetitive behaviors [14,15]. Pramipexole has a selective affinity for dopamine D3 receptors [16,17], which are primarily confined to the limbic system [18], and might prime the dopamine reward circuits thus facilitating emergence of compulsive behaviors. Moreover, music listening has been correlated with a significant activation of the ventral tegmental area and accumbens nucleus, as well as of the hypothalamus, insula, and orbitofrontal cortex [19–21]. These findings suggest that music listening may recruit similar neural circuitry of reward and emotions as other pleasure inducing stimuli, and this may also be the case for playing instruments.

Unusual repetitive behaviors could be common and unrecognized complications associated with dopamine agonists use in PD. Physicians should be alert to the possibility of such striking behaviors, including CD, when administering a dopamine agonist in PD patients.
